# Online Clinical Calculator for Predicting 28-Day Mortality in Older Adult Patients With Sepsis-Associated Encephalopathy: Retrospective Study Using MIMIC-IV

**DOI:** 10.2196/76417

**Published:** 2025-12-04

**Authors:** Guangyong Jin, Menglu Zhou, Jiayi Chen, Mengyuan Diao, Wei Hu

**Affiliations:** 1 Department of Critical Care Medicine Affiliated Hangzhou First People’s Hospital, School of Medicine, Westlake University Hangzhou China; 2 Department of Critical Care Medicine Hangzhou Geriatric Hospital Hangzhou China; 3 Department of Rehabilitation Affiliated Hangzhou First People’s Hospital, School of Medicine, Westlake University Hangzhou China

**Keywords:** clinical prediction model, geriatrics, intensive care unit, outcome assessment, sepsis-associated encephalopathy

## Abstract

**Background:**

Sepsis-associated encephalopathy (SAE) represents a critical complication of sepsis, especially among older adults. Despite its clinical relevance, there remains a lack of accessible and practical tools specifically designed to predict 28-day mortality in this vulnerable population.

**Objective:**

We aimed to enhance the practical applicability of the model by creating a web-based tool that allows real-time, individualized mortality risk prediction, facilitating early intervention and informed decision-making in clinical practice.

**Methods:**

Using data extracted from the MIMIC-IV (Medical Information Mart for Intensive Care IV) database, we identified older patients (≥65 years) with SAE (n=2165) and divided them into a development cohort (n=1531) and a validation cohort (n=634). Key risk factors associated with 28-day mortality were identified, and a predictive nomogram was constructed. Model performance was evaluated using the concordance index, integrated discrimination improvement, net reclassification index, and calibration curve analysis. Clinical applicability was assessed through decision curve analysis and benchmarked against traditional intensive care unit (ICU) scoring systems. Furthermore, the nomogram was deployed as a web-based application, enabling clinicians to input data and generate individualized mortality predictions.

**Results:**

A total of 2165 older patients with SAE were included, among whom 290 (13.4%) died within 28 days of ICU admission. Multivariable logistic regression identified lower body weight (odds ratio [OR] 0.985, 95% CI 0.975-0.994; *P*=.001), lower systolic blood pressure (OR 0.972, 95% CI 0.957-0.986; *P*<.001), lower hemoglobin (OR 0.984, 95% CI 0.974-0.995; *P*=.005), lower PaO2 (OR 0.996, 95% CI 0.994-0.997; *P*<.001), and lower Glasgow Coma Scale score (OR 0.825, 95% CI 0.786-0.864; *P*<.001) as mortality risk factors. Higher respiratory rate (OR 1.083, 95% CI 1.029-1.141; *P*=.002), increased anion gap (OR 1.081, 95% CI 1.031-1.135; *P*=.001), elevated blood urea nitrogen (OR 1.045, 95% CI 1.016-1.076; *P*=.002), prolonged partial thromboplastin time (OR 1.033, 95% CI 1.016-1.050; *P*<.001), and reduced urine output (OR>0.99, 95% CI 0.999-1.000; *P*=.002) were also predictive. Patients admitted to “other” ICU types had lower mortality compared with the medical ICU reference group (OR 0.327, 95% CI 0.176-0.609; *P*<.001). The nomogram achieved concordance index values of 0.899 (development) and 0.897 (validation), outperforming sequential organ failure assessment (0.692), Acute Physiology Score III (0.804), Logistic Organ Dysfunction System (0.771), Simplified Acute Physiology Score II (0.704), and Oxford Acute Severity of Illness Score (0.753), with significant integrated discrimination improvement and net reclassification index improvements (all *P*<.001). Calibration curves confirmed good agreement between predicted and observed outcomes, while decision curve analysis supported the model’s superior clinical utility.

**Conclusions:**

This study presents a novel, validated nomogram for predicting 28-day mortality in older patients with SAE, integrating routinely available clinical data. The deployment of the model as a digital tool enhances its accessibility and usability, providing clinicians with a practical resource for risk stratification and individualized patient management.

## Introduction

Sepsis stands as a prevalent critical condition encountered within intensive care units (ICUs), presenting a formidable obstacle to global health care systems [1,2]. Characterized by diffuse cerebral dysfunction devoid of direct central nervous system infection or structural anomalies, sepsis-associated encephalopathy (SAE) emerges as a significant complication of sepsis [3]. The escalating global phenomenon of population aging has further accentuated the burden of sepsis [4-6], with a disproportionately heightened incidence observed among the older adult demographic [7]. Advanced age, in itself, emerges as an independent predictor of sepsis mortality, with older adult nonsurvivors succumbing earlier during hospitalization compared to their younger counterparts [7]. Nevertheless, older adult survivors of sepsis frequently necessitate specialized nursing or rehabilitative care post discharge [7]. Moreover, within the realm of older adult sepsis patients, the risk of developing SAE amplifies with advancing age, concomitantly exacerbating prognosis [6]. Precise prognostication of older adult patients with SAE holds paramount importance, facilitating risk stratification, fostering effective doctor-patient communication, and guiding clinical decision-making and therapeutic interventions.

The mortality prediction model, as a fundamental prognostic tool, epitomizes a distinct subset within clinical prediction models. Operating on the principle of predicting the unknown through the known, these models embody mathematical formulations wherein known variables are leveraged to compute the probability of an unforeseen outcome [8,9]. Central to the efficacy of such models lie the predictive variables, which serve as the bedrock for their construction [10]. When deliberating variable selection, considerations spanning economic feasibility, standardization, and accessibility necessitate careful scrutiny [10]. Conventional clinical parameters and hematological indices emerge as prime candidates for inclusion, owing to their cost-effectiveness, standardized measurement protocols, and ease of acquisition, thus positioning them as pivotal elements in the construction of robust clinical prediction models.

While several predictive models have been devised to anticipate mortality rates in cases of SAE [11-14], the development of such models tailored specifically for the older adult cohort remains, to the best of our knowledge, unprecedented. Therefore, this study aimed to develop and validate a clinically useful nomogram based on readily available clinical data from the MIMIC-IV (Medical Information Mart for Intensive Care IV) database. Additionally, we aimed to enhance the practical applicability of the model by creating a web-based tool that allows real-time, individualized mortality risk prediction, facilitating early intervention and informed decision-making in clinical practice.

## Methods

### Database

In adherence to the TRIPOD (Transparent Reporting of a Multivariable Prediction Model for Individual Prognosis or Diagnosis) guidelines [9], this retrospective investigation harnessed data derived from the MIMIC-IV database, version 2.1, for the purpose of clinical predictive model development. MIMIC-IV serves as a repository for an extensive array of clinical data sourced from tens of thousands of patients treated at the Beth Israel Deaconess Medical Center in Boston, Massachusetts, encompassing a spectrum of vital signs, hematological parameters, and therapeutic interventions [15]. The primary investigator has undergone requisite human research training (certificate number 46141344) and has executed a PhysioNet-certified health data usage agreement, thereby obtaining authorized access to the MIMIC-IV database.

### Ethical Considerations

The Institutional Review Committee at Beth Israel Deaconess Medical Center meticulously scrutinized the process of patient data collection and the establishment of the MIMIC (Medical Information Mart for Intensive Care) database [16]. Subsequently, the committee accorded an exemption from the requirement of obtaining informed consent and provided explicit approval for the dissemination of data as per the predefined data sharing protocol. In adherence to stringent privacy standards, a randomized cipher was used to substitute patient identifiers, thereby generating deidentified integer codes for patients, hospitalizations, and ICU admissions [16].

In accordance with prevailing national regulations governing research involving human subjects in life sciences and medicine, studies using human information data devoid of potential harm to individuals, sensitive personal details, or commercial interests may qualify for exemption from ethical review. Specifically, such exemptions apply to research endeavors using legally acquired public data or anonymized information. Our study satisfies the stipulated criteria for exemption from ethical review. Moreover, given the retrospective nature of our investigation, the requirement for individual informed consent has been waived in accordance with prevailing national regulations. No compensation was provided to participants.

### Study Participants

The study cohort comprises older adult patients with SAE undergoing their initial admission to the ICU. Inclusion criteria were ascertained as follows [3,6,11-14]: (1) patients classified as sepsis 3.0, denoting those diagnosed or suspected of infection within the MIMIC database with a SOFA (sequential organ failure assessment) score of ≥2. The MIMIC code library provides comprehensive codes for sepsis determination [17]; (2) SAE identification criteria: sepsis patients were deemed to have SAE if they met any of the following conditions on the first day after ICU admission: Glasgow Coma Scale (GCS) score=15 points with delirium, or GCS score <15 points. Delirium was determined based on positive Confusion Assessment Method for the Intensive Care Unit, or confirmed *ICD* (*International Classification of Diseases*) codes (2930, 2931, F05); (3) older adult classification: patients aged between 65 and 89 years (MIMIC encodes patients older than 89 years as 91 for deidentification purposes).

**Figure 1:**
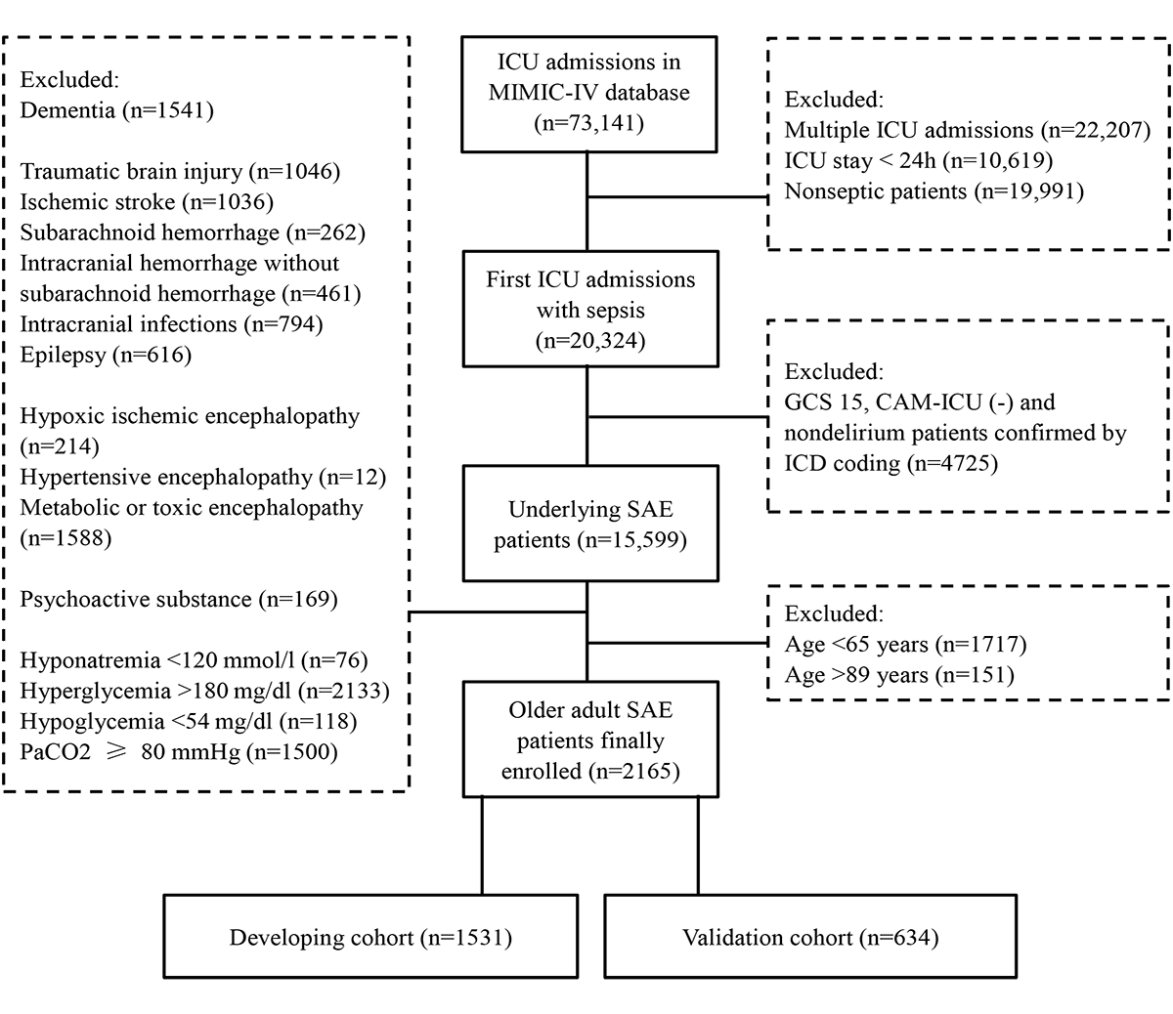
Diagrammatic illustration of the patient selection protocol. CAM-ICU: Confusion Assessment Method for the Intensive Care Unit; GCS: Glasgow Coma Scale; ICD: International Classification of Diseases; ICU: intensive care unit; MIMIC-IV: Medical Information Mart for Intensive Care IV; PaCO2: partial pressure of CO2; SAE: sepsis-associated encephalopathy.

Exclusion criteria were formulated based on prior investigations [11,13,14], comprising: (1) records indicating second or subsequent ICU admissions; (2) ICU duration less than 24 hours; (3) diagnosis of dementia; (4) primary brain injuries, inclusive of traumatic brain injury, ischemic stroke, subarachnoid hemorrhage, intracranial hemorrhage excluding subarachnoid hemorrhage, intracranial infections, and epilepsy; (5) hypoxic ischemic encephalopathy; (6) hypertensive encephalopathy; (7) metabolic or toxic encephalopathy; (8) mental and behavioral disorders attributed to alcohol, drug, or psychoactive substance usage, including delirium induced by opioids, cannabis, cocaine, sedatives, hallucinogens, or other psychoactive substances; (9) concurrent severe electrolyte or blood glucose disturbances, such as hyponatremia (<120 mmol/L), hyperglycemia (>180 mg/dl), or hypoglycemia (<54 mg/dl) and PaCO2 (partial pressure of CO2) ≥ 80 mm Hg. The process of cohort selection is outlined in Figure 1.

### Data Extraction and Preparation

Data preparation and statistical processing in this study closely mirror methodologies used in our prior investigations [15,18]. Extracted research data encompass demographic particulars such as age, gender, weight, marital status, race, underlying medical conditions, and Charlson Comorbidity Index scores. Additionally, vital signs, pivotal laboratory parameters, use of invasive mechanical ventilation, renal replacement therapy, administration of vasoactive agents, albumin infusion, GCS scores, and other critical care scoring metrics were captured on the inaugural day of ICU admission. Vasoactive drug use encompassed the administration of norepinephrine, adrenaline, dopamine, dobutamine, vasopressin, or milrinone. For variables measured multiple times on the initial day of ICU admission, MIMIC concepts were used to derive minimum, maximum, and, if applicable, average values.

The primary end point of interest was the 28-day mortality, ascertained based on survival status 28 days post-ICU admission.

Preliminary dataset preparation entailed outlier management and handling of missing values, executed using Stata software (version 17.0, Stata Corporation LLC). As illustrated in Figure S1 of Multimedia Appendix 1, the extent of missingness for each variable was visualized using the *DataExplorer* package in R (version 4.3.2, R Foundation for Statistical Computing). Variables with more than 20% missing values were excluded from further analysis to ensure data quality. Initial outlier detection entailed histogram analysis to pinpoint anomalies within the dataset. Subsequent to identification, winsorization was used to mitigate outlier influence by replacing extreme values with thresholds established at the 0.1% and 99.9% quantiles using the Winsor2 command. Addressing missing data conundrums necessitated the application of diverse interpolation techniques to generate 5 sets of interpolated data. For variables with ≤20% missingness, multiple imputation was performed using Stata under the multivariate normal regression framework, as implemented by the command. The imputation model included all candidate predictor variables, relevant demographic characteristics, comorbidities, and severity scores to preserve associations among variables and reduce bias. Five imputed datasets were generated with 500 iterations of Markov Chain Monte Carlo data augmentation following a 100-iteration burn-in phase. Collinearity was checked before imputation, and variables causing collinearity were omitted automatically by Stata. Post imputation, 1 randomly selected dataset was extracted for subsequent analyses, following Rubin rules for ensuring consistency across imputations. Descriptive checks confirmed that imputed values were within plausible clinical ranges and maintained the overall distributional properties of the observed data.

### Statistical Analysis

Statistical analyses were conducted using R software (version 4.3.2). The cohort of older adult patients with SAE was randomly partitioned into development and validation subsets at a ratio of 7:3, using a seed size of 73. Univariate analyses were performed on both the development and validation cohorts using the “gtsummary” software package. Continuous variables demonstrating nonnormal distribution were summarized using median and IQR, while categorical variables were presented as numerical counts or percentages. Statistical significance was defined as *P* value <.05.

In the development cohort, LASSO (least absolute shrinkage and selection operator) regression, facilitated by the “glmnet” R package, was used to identify potential variables associated with 28-day mortality in older adult patients with SAE. Subsequently, multiple-factor binary logistic regression was conducted to discern the final independent risk factors. The “car” R package was used to compute the variance inflation factor, assessing collinearity between variables. A prognostic nomogram was constructed using the “rms” R data package.

To evaluate the predictive performance of the nomogram, the “pROC” R package was used to calculate the concordance index (C-index), while the “PredictABEL” R software package facilitated computation of the integrated discrimination improvement (IDI) and net reclassification improvement (NRI). Receiver operating characteristic curves for both the development and validation datasets were generated using the “riskRegression” R package to assess model sensitivity and specificity across different probability thresholds. Calibration of the nomogram was assessed via generation of a calibration curve using the “val.prob” function within the “rms” R data package. Additionally, the Hosmer-Lemeshow goodness-of-fit test was performed using the “hoslem.test” function in the *ResourceSelection* R package to further evaluate model calibration. Subsequently, decision curve analysis was conducted using the “rmda” R data package to meticulously evaluate the clinical utility and benefits conferred by the developed nomogram. We deployed the developed predictive model as a web-based tool using the *Shiny* and *rsconnect* packages in R. This web-based application allows users to input patient-specific data and obtain an estimated probability of 28-day mortality, facilitating clinical utility and accessibility of the nomogram.

## Results

### Baseline Characteristics

A total of 2165 older adult patients with SAE were included, among whom 290 (13.4%) died within 28 days of ICU admission. Table S1 in Multimedia Appendix 2 offers a comprehensive summary of the baseline characteristics observed across the entire cohort of older adult patients with SAE, delineating findings from both the developmental cohort (n=1531) and the validation cohort (n=634). Following meticulous examination, no statistically significant differences (*P*>.05) were discerned in demographic attributes, comorbidities, indices of disease severity, vital signs, common hematological parameters, or therapeutic interventions between the development and validation cohorts. Notably, the absence of statistically significant distinctions underscores the efficacy of random allocation methodologies in ensuring comparability between cohorts, thus underscoring the robust scientific underpinnings guiding cohort allocation. Older adult patients with SAE were stratified into distinct survival and mortality groups based on their 28-day survival outcomes. Baseline comparisons between these groups within the development and validation cohorts are presented in the supplementary materials for comprehensive elucidation.

### Variable Selection and Nomogram Construction

**Figure 2:**
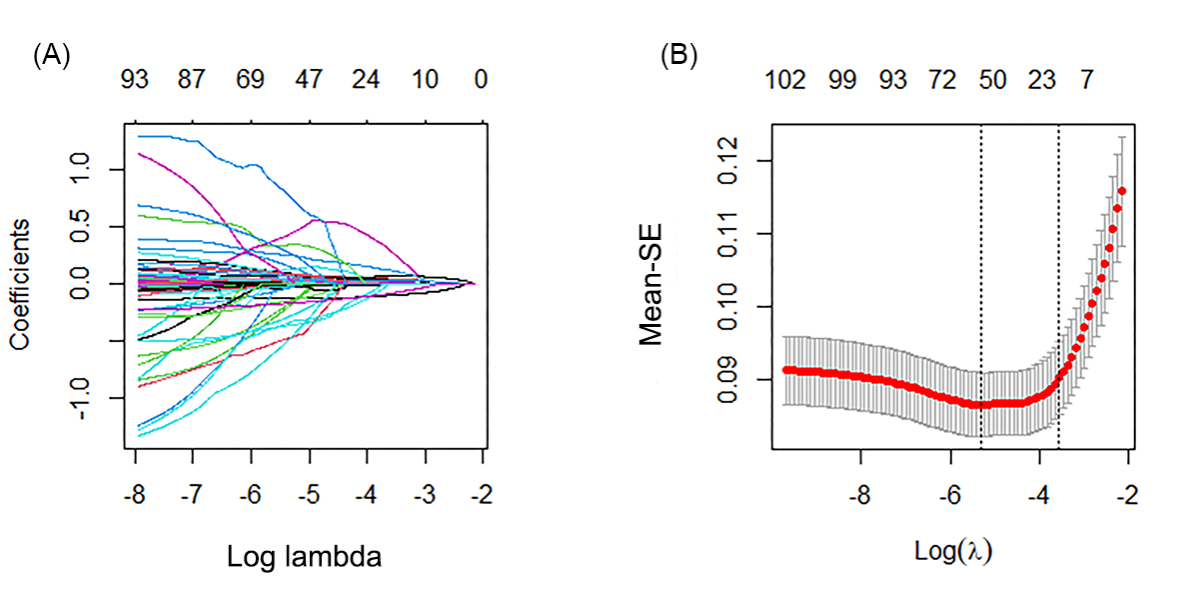
LASSO regression-based feature selection process with tenfold cross-validation. (A) The plot illustrates the relationship between the coefficients of clinical features and lambda values within the LASSO regression framework. (B) The graph depicts the 10-fold cross-validation curve used in LASSO regression, offering insights into model selection. The left dashed vertical line indicates the optimal log (lambda) value (λ=0.004900582) corresponding to the minimum mean SE. Additionally, following the 1 SE rule, the right dashed vertical line represents the model incorporating 11 variables (λ=0.02870281). LASSO: least absolute shrinkage and selection operator regression.

**Figure 3:**
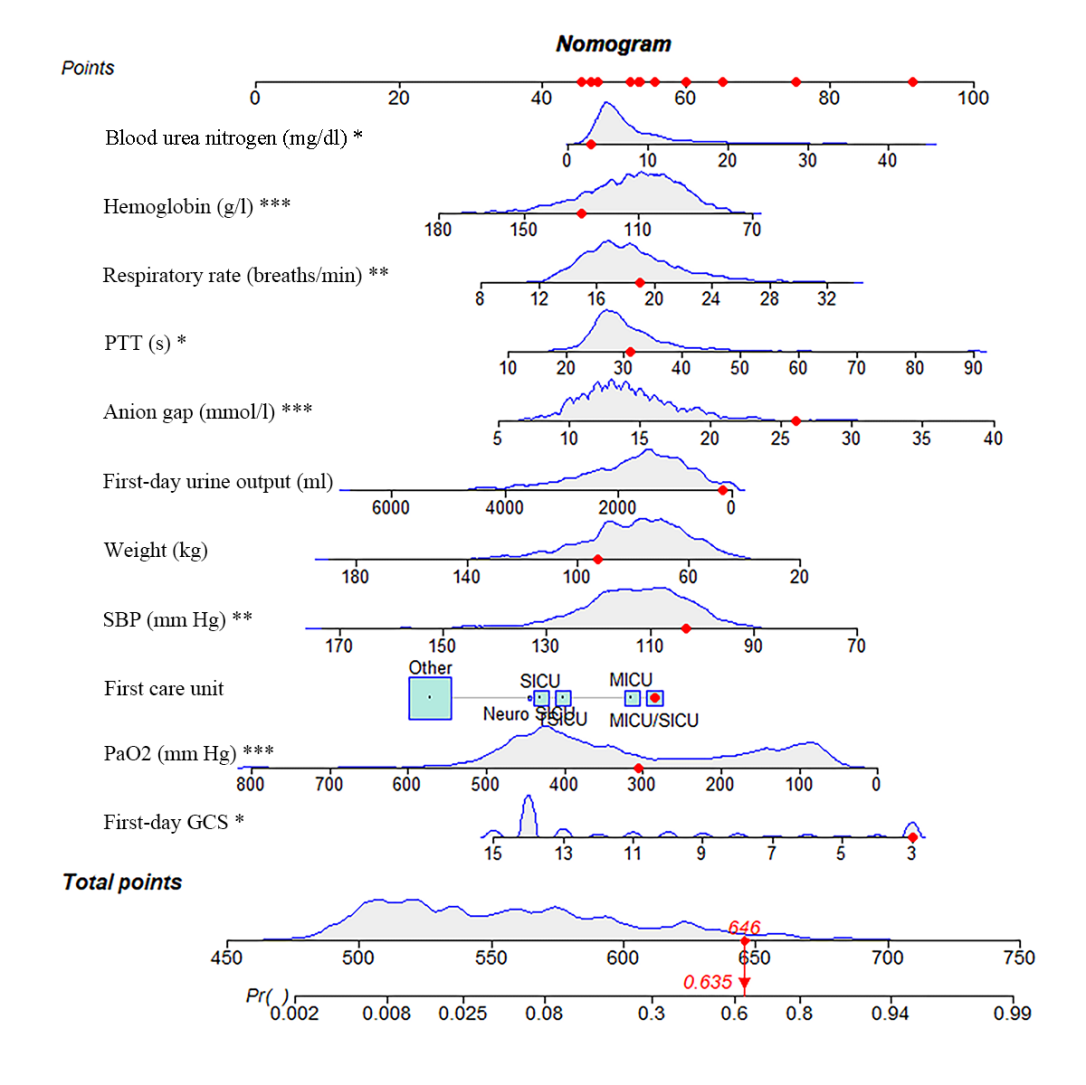
Nomogram visualization for predicting 28-day mortality probability in geriatric patients with SAE. This graphical representation illustrates a nomogram tailored to estimate the likelihood of 28-day mortality among older adult individuals diagnosed with SAE. The nomogram operates by assigning points to specific variables based on their respective values. These points are then totaled, and the corresponding 28-day mortality rate can be determined by referencing the total points axis. This depiction elucidates the operational framework of the nomogram in forecasting personalized mortality risks for older adult patients with SAE. *Represents the minimum recorded values of essential indicators observed on the initial day of admission to the intensive care unit. **Indicates the mean values of clinical parameters recorded within the first day of intensive care unit admission. ***Denotes the maximum values of relevant indicators observed during the initial day of intensive care unit admission. GCS: Glasgow Coma Scale; MICU: medical intensive care unit; Neuro SICU: neurosurgical intensive care unit; PTT: partial thromboplastin time; SAE: sepsis-associated encephalopathy; SBP: systolic blood pressure; SICU: surgical intensive care unit; TSICU: trauma surgical intensive care unit.

Using a hybrid approach of LASSO regression coupled with 10-fold cross-validation (Figure 2A and 2B), potential clinical determinants correlating with 28-day mortality in older adult patients with SAE were identified. Subsequent binary multivariate logistic regression analysis corroborated the significance of 11 distinct clinical factors or hematological parameters as independent predictors of 28-day mortality within this cohort (*P*<.05; Table 1). All included predictors demonstrated acceptable collinearity, with variance inflation factor values presented in Multimedia Appendix 3 (Figure S1), confirming that multicollinearity did not compromise the stability of the regression model. These discerned variables were subsequently integrated to devise a prognostic nomogram (Figure 3).

**Table 1:** Multivariable logistic regression analysis reveals independent predictors of 28-day mortality in older adult patients with sepsis-associated encephalopathy: findings from the developmental cohort.

Variables	OR^a^ (95% CI)	*P* value
Weight (kg)	0.985 (0.975-0.994)	.001
First care unit (SICU^b^)	0.605 (0.305-1.177)	.14
First care unit (MICU^c^/SICU)	1.135 (0.635-2.033)	.67
First care unit (Neuro SICU^d^)	0.567 (0.027-4.650)	.64
First care unit (TSICU^e^)	0.683 (0.344-1.332)	.27
First care unit (Other)	0.327 (0.176-0.609)	<.001
SBP^f^ (mm Hg)^g^	0.972 (0.957-0.986)	<.001
Respiratory rate (breaths/min)^g^	1.083 (1.029-1.141)	.002
First-day urine output (ml)	1.000 (0.999-1.000)	.002
Hemoglobin (g/l)^h^	0.984 (0.974-0.995)	.005
Anion gap (mmol/l)^h^	1.081 (1.031-1.135)	.001
Blood urea nitrogen (mg/dl)^i^	1.045 (1.016-1.076)	.002
PTT^j^ (s)^i^	1.033 (1.016-1.050)	<.001
PaO2 (mm Hg)^h^	0.996 (0.994-0.997)	<.001
First-day GCS^i,k^	0.825 (0.786-0.864)	<.001

^a^OR: odds ratio.

^b^SICU: surgical intensive care unit.

^c^MICU: medical intensive care unit.

^d^Neuro SICU: neurosurgical intensive care unit.

^e^TSICU: trauma surgical intensive care unit.

^f^SBP: systolic blood pressure.

^g^Denotes the mean values of relevant indicators observed during the initial day of intensive care unit admission.

^h^Indicates the maximum values of clinical parameters recorded within the first day of intensive care unit admission.

^i^Represents the minimum recorded values of essential indicators observed on the initial day of admission to the intensive care unit.

^j^PTT: partial thromboplastin time.

^k^GCS: Glasgow Coma Scale.

Figure 3 depicts a representative case: an older adult patient with SAE, weighing 93 kg, was admitted to the medical or surgical intensive care unit (SICU). On the initial day of admission, the patient exhibited the following clinical and hematological parameters: the lowest blood urea nitrogen level recorded was 2.86 mg/dl, the highest hemoglobin concentration was 130 g/l, the mean respiratory rate was 18.96 beats/min, the lowest partial thromboplastin time (PTT) was 31.1 seconds, the maximum anion gap (AG) was 26 mmol/L, the first day urine output was 185 ml, the average systolic blood pressure was 103.02 mm Hg, the highest partial pressure of arterial oxygen was 306 mm Hg, and the lowest GCS score was 3 points. Based on these findings, the cumulative score for this patient was calculated as 646 points, corresponding to a predicted 28-day mortality rate of 0.635.

### Discriminative Performance and Calibration Assessment of Nomogram

The derived prognostic nomogram exhibited a calculated C-index of 0.899 (95% CI 0.877-0.921) in the development cohort and 0.897 (95% CI 0.864-0.929) in the validation cohort. Comparative analyses revealed that the discriminatory performance of the constructed nomogram surpassed conventional benchmarks, including GCS score and commonly used disease severity scoring systems (Table 2). Receiver operating characteristic curve analysis was also performed to assess model sensitivity and specificity across different thresholds, with the resulting curves for both the development and validation datasets presented in Figure S1 of Multimedia Appendix 4, further demonstrating the classification ability and clinical utility of the nomogram. Furthermore, evaluations using IDI and NRI metrics revealed substantial enhancements in the predictive accuracy of the devised nomogram for 28-day mortality (both *P*<.001; Table 3).

**Table 2:** Assessment of C-index^a^ performance: nomogram versus models incorporating commonly used ICU^b^ scoring systems for predicting 28-day mortality in geriatric patients with sepsis-associated encephalopathy.

Models	Development dataset, C-index (95% CI)	Validation dataset, C-index (95% CI)
Nomogram	0.899 (0.877-0.921)	0.897 (0.864-0.929)
GCS^c^	0.664 (0.626-0.702)	0.603 (0.537-0.668)
SOFA^d^	0.692 (0.652-0.732)	0.715 (0.654-0.776)
APS III^e^	0.804 (0.775-0.832)	0.783 (0.733-0.833)
LODS^f^	0.771 (0.736-0.806)	0.746 (0.686-0.806)
SAPS II^g^	0.704 (0.667-0.741)	0.718 (0.660-0.777)
OASIS^h^	0.753 (0.718-0.789)	0.741 (0.681-0.800)

^a^C-index: concordance index.

^b^ICU: intensive care unit.

^c^GCS: Glasgow Coma Scale.

^d^SOFA: sequential organ failure assessment.

^e^APS III: Acute Physiology Score III.

^f^LODS: Logistic Organ Dysfunction System.

^g^SAPS II: Simplified Acute Physiology Score II.

^h^OASIS: Oxford Acute Severity of Illness Score.

**Table 3:** Evaluation of NRI^a^ and IDI^b^ in predictive models for 28-day mortality among geriatric patients afflicted with sepsis-associated encephalopathy.

Index^c^	Development dataset			Validation dataset	
	Estimate (95% CI)	*P* value	Estimate (95% CI)		*P* value
NRI (vs GCS^d^)	0.709 (0.624-0.795)	<.001	0.651 (0.531-0.771)		<.001
NRI (vs SOFA^e^)	0.576 (0.487-0.666)	<.001	0.405 (0.267-0.543)		<.001
NRI (vs APS III^f^)	0.398 (0.309-0.488)	<.001	0.339 (0.205-0.472)		<.001
NRI (vs LODS^g^)	0.405 (0.309-0.501)	<.001	0.359 (0.213-0.506)		<.001
NRI (vs SAPS II^h^)	0.585 (0.499-0.670)	<.001	0.570 (0.432-0.708)		<.001
NRI (vs OASIS^i^)	0.435 (0.335-0.536)	<.001	0.346 (0.189-0.502)		<.001
IDI (vs GCS)	0.325 (0.289-0.361)	<.001	0.324 (0.268-0.379)		<.001
IDI (vs SOFA)	0.287 (0.252-0.321)	<.001	0.240 (0.191-0.288)		<.001
IDI (vs APS III)	0.183 (0.150-0.216)	<.001	0.186 (0.143-0.228)		<.001
IDI (vs LODS)	0.213 (0.178-0.247)	<.001	0.215 (0.160-0.269)		<.001
IDI (vs SAPS II)	0.293 (0.258-0.328)	<.001	0.271 (0.217-0.326)		<.001
IDI (vs OASIS)	0.240 (0.202-0.278)	<.001	0.226 (0.170-0.282)		<.001

^a^NRI: net reclassification index.

^b^IDI: integrated discrimination improvement.

^c^Cutoff: 0, 0.2, 0.4, 1.

^d^GCS: Glasgow Coma Scale.

^e^SOFA: sequential organ failure assessment.

^f^APS III: Acute Physiology Score III.

^g^LODS: Logistic Organ Dysfunction System.

^h^SAPS II: Simplified Acute Physiology Score II.

^i^OASIS: Oxford Acute Severity of Illness Score.

**Figure 4:**
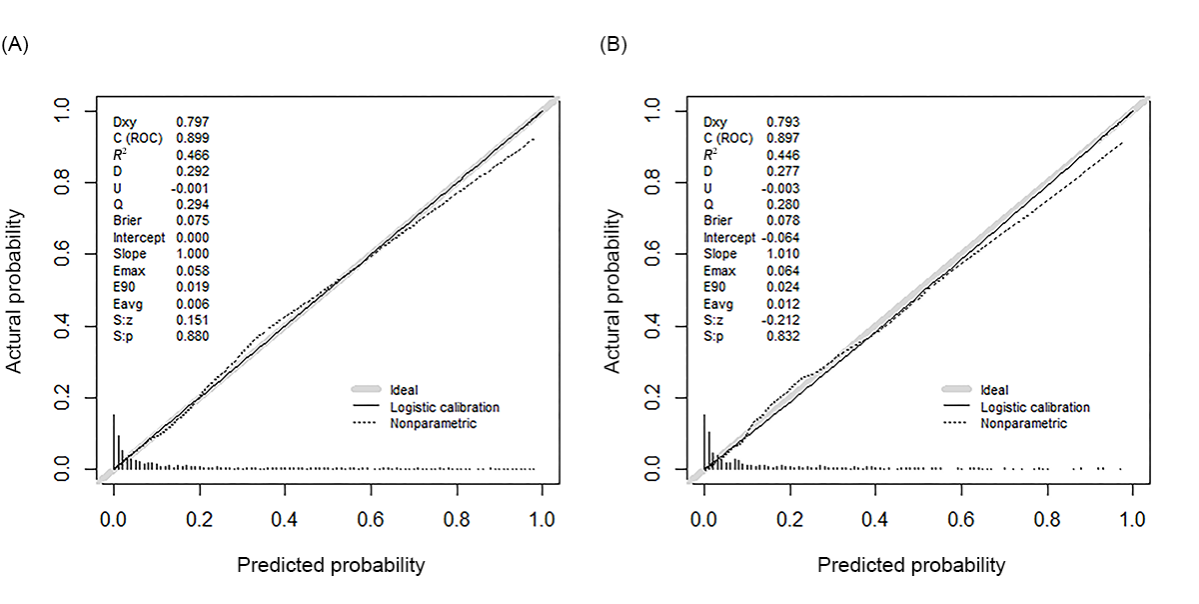
Calibration curve assessing the predictive accuracy of the established nomogram for 28-day mortality. This calibration curve illustrates the alignment between predicted and observed 28-day mortality rates in both the (A) development and (B) validation sets. The lack of statistically significant differences (*P*>.05) indicates the robustness and reliability of the predictive model, affirming its accuracy in estimating mortality outcomes. C (ROC): concordance index (receiver operating characteristic curve); D: discrimination index; Dxy: rank correlation between predicted probabilities and observed values; E90: 90th percentile of the prediction error; Eavg: average error; Emax: maximum absolute error; Q: quality index; R2: Nagelkerke-Cox-Snell-Maddala-Magee R-squared index; S:p: the *P* value of Spiegelhalter z-test; S:z: The z-value of Spiegelhalter z-test; U: unreliability index.

Significantly, a striking resemblance between the anticipated and observed 28-day mortality rates was observed, with statistical analyses revealing no discernible differences within both the development and validation cohorts (Figure 4A and 4B). The Hosmer-Lemeshow goodness-of-fit test further confirmed adequate calibration of the nomogram, with nonsignificant results in both the development cohort (*χ²*_8_=4.6, *P*=.81) and the validation cohort (*χ²*_8_=8.7, *P*=.37), indicating no evidence of lack of fit.

### Evaluation of Nomogram Clinical Applicability in Predicting 28-Day Mortality in Older Adult Patients With SAE

**Figure 5:**
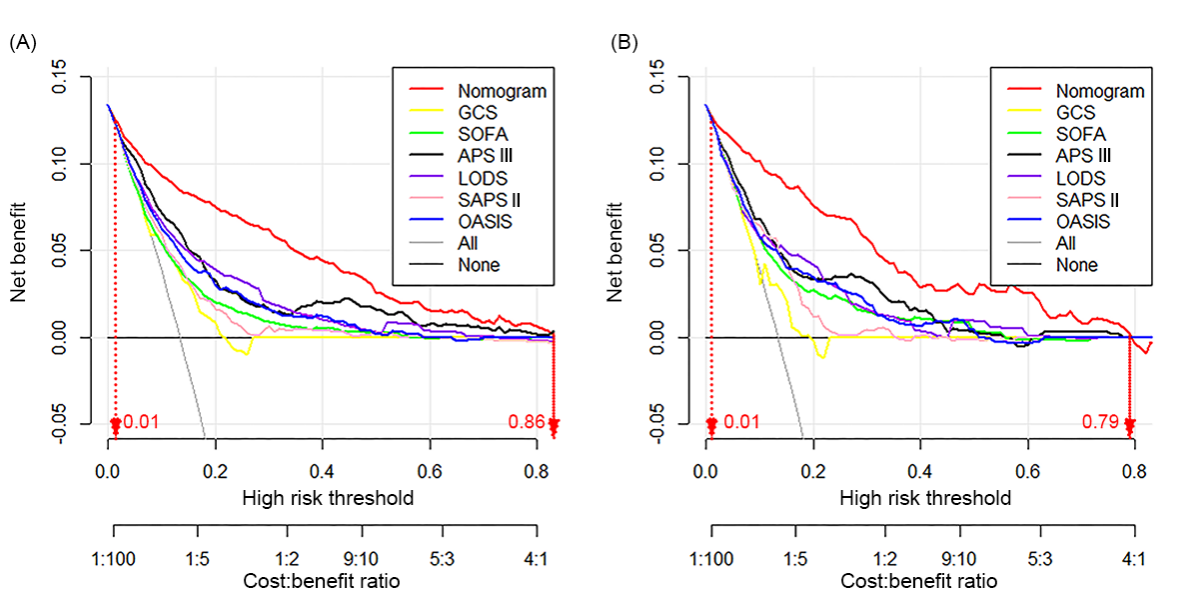
Evaluation of clinical utility through decision curve analysis: comparative assessment between the developed nomogram and established clinical scoring systems in the training and validation cohorts. Decision curve analysis is used to compare the clinical utility of the nomogram developed in this study with conventional clinical scoring systems in predicting outcomes. The red line represents the nomogram, which showed higher net benefit across the threshold ranges of 0.01-0.86 (training cohort) and 0.01-0.79 (validation cohort). APS III: Acute Physiology Score III; GCS: Glasgow Coma Score; LODS: Logistic Organ Dysfunction System; OASIS: Oxford Acute Severity of Illness Score; SAPS II: Simplified Acute Physiology Score II; SOFA: sequential organ failure assessment.

As depicted in Figure 5, the graphical representation illustrates the pronounced net clinical benefit conferred by the developed nomogram, denoted by the red line, in comparison to established metrics such as the GCS score and widely used scoring systems. Across a probability threshold range of 0.01-0.86 in the development cohort and 0.01-0.79 in the validation cohort, the nomogram consistently demonstrated greater net benefit. Remarkably, our formulated nomogram demonstrates markedly heightened clinical effectiveness, underscoring its superior practical applicability and utility in prognostication relative to conventional scoring methodologies.

### Deployment of the Web-Based Nomogram

To enhance clinical applicability, we deployed the developed nomogram as a web-based calculator using the *Shiny* and *rsconnect* packages in R. This web-based tool is accessible online [19]. Clinicians can input readily available clinical parameters to obtain an individualized estimate of 28-day mortality risk in older adult patients with SAE.

**Figure 6:**
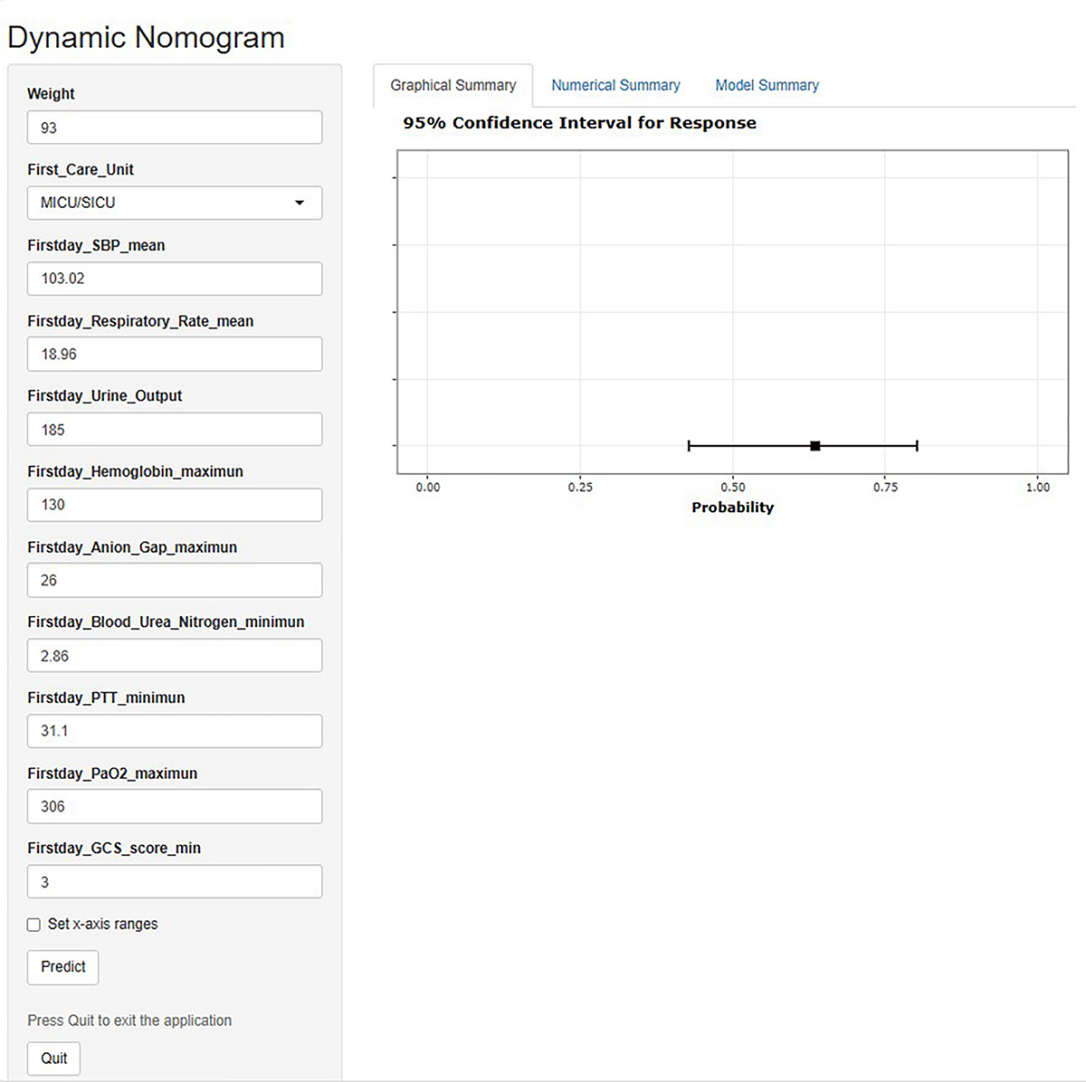
Web-based application demonstration using a representative patient case. GCS: Glasgow Coma Scale; MICU: medical intensive care unit; SBP: systolic blood pressure; SICU: surgical intensive care unit; PTT: partial thromboplastin time.

To illustrate the practical use of the web-based calculator, we applied the same patient case presented in Figure 3 to generate a predicted probability of 28-day mortality through the web interface (Figure 6). As shown in Figure 6, the model provides an intuitive visual output that complements the static nomogram, offering immediate clinical insight for bedside decision-making.

The web-based dynamic calculator interface for the 28-day mortality nomogram is shown. Clinical parameters from the same representative case in Figure 3 were entered into the web tool, generating an individualized prediction of 28-day mortality risk. The tool provides an intuitive output that enhances the model’s accessibility and facilitates clinical application at the bedside.

## Discussion

### Principal Results

In the context of societal aging, precise evaluation of short-term prognosis among older adult patients with SAE becomes imperative. Regrettably, the current landscape lacks tailored clinical prediction models for this demographic subset. To our knowledge, our study marks the inaugural development and validation of a clinical prediction model meticulously crafted to forecast 28-day mortality rates in older adult patients with SAE. Moreover, the deployment of the nomogram as a web-based application enhances its accessibility and ease of use, offering real-time, individualized mortality risk assessments that can support decision-making in busy clinical settings.

Clinical prediction models for SAE have attracted increasing academic interest, with existing studies mainly focusing on 2 aspects: models predicting the occurrence of SAE and models predicting mortality among patients with SAE [11-14,20-23]. For example, a nomogram was constructed using 9 variables identified through multivariable regression analysis to predict the occurrence of SAE. However, it is noteworthy that the use of midazolam was also included as one of the predictive factors, which may be somewhat perplexing, because alterations in consciousness induced by midazolam require careful distinction from those attributable to SAE [22]. Older adult populations have also received special attention, as SAE has been identified as an independent risk factor for in-hospital mortality among older adult ICU patients, and a nomogram has been established to predict the probability of SAE in this subgroup [21]. Machine learning methods have likewise been applied to SAE prediction, with 6 classifiers compared for their performance in predicting SAE occurrence. Among them, an Extreme Gradient Boosting–based model demonstrated superior predictive accuracy using simple clinical indicators while offering visual interpretability [23]. However, this model relied on predictors such as length of stay and ventilation duration [23]. In practice, such time-dependent indicators may only become available late in the hospitalization process, at which point SAE onset may already be apparent, thus limiting clinical utility.

Prediction models have also been developed for SAE-related mortality, using both nomograms and machine learning methods to estimate 30-day outcomes [11,13]. For instance, models using APS III (Acute Physiology Score III) and SOFA score as predictors have been reported [13], though their clinical applicability is hampered by the complexity of APS III calculation. More advanced machine learning approaches have even combined the 3 best-performing classifiers (elastic net, support vector machine, and Extreme Gradient Boosting) into a stacked ensemble model for predicting ICU mortality in patients with SAE. This model achieved an internal validation AUC of 0.807, but external validation revealed a reduced AUC of 0.671, raising concerns about generalizability and stability [20]. In comparison with extant clinical prediction models for SAE [11-14,20], our study shares a commonality in the inclusion of GCS scores and respiratory rate as key independent variables. This convergence offers novel avenues for future investigations into SAE-related prediction models. However, our investigation diverges by specifically targeting SAE mortality rates in the older adult population [11-14], leveraging the updated MIMIC-IV database. Notably, meticulous attention was devoted to refining inclusion and exclusion criteria for patients with SAE during data processing, ensuring the enrollment of cohorts with comprehensive data profiles and mitigating selection biases. Using the simplest supervised learning algorithm predicated on binary classification outcomes, our study used logistic regression, showcasing distinct advantages over alternative machine learning methodologies [8,24,25]. Notable benefits include a parsimonious model structure, robust fitting, and enhanced user-friendliness. Despite its simplicity, the nomogram devised in our study incorporates a select number of variables yet demonstrates commendable discriminative capacity. Furthermore, the nomogram’s accessibility renders it amenable to integration into routine clinical practice, bolstering its utility in real-world health care settings.

Leveraging commonplace clinical data and hematological parameters sourced from the MIMIC-IV database, our model stands as a user-friendly tool poised to address this critical gap. Using LASSO regression for initial dimensionality reduction mitigates overfitting concerns and enhances model practicality and interpretability [26,27]. Logistic regression, a venerable statistical technique in clinical research, aids in identifying independent risk factors [28]. Our analysis pinpointed several variables, including type of first care unit, GCS score, weight, systolic blood pressure, respiratory rate, urine output, and hematological indices (blood urea nitrogen, hemoglobin, PTT, AG, and PaO2), as significant predictors. Evaluation metrics such as the C-index gauge predictive efficacy across development and validation cohorts [29], while NRI and IDI analyses highlight performance enhancements relative to conventional ICU scoring systems. Calibration curves attest to the alignment of predicted outcomes with actual observations. Furthermore, decision curve analysis underscores the superior practical utility and clinical decision-making benefits of our predictive model compared to existing scoring systems. Additionally, a user-friendly column chart has been devised for seamless clinical integration, thus facilitating wider adoption and dissemination of our model in clinical practice.

The GCS score stands as a pragmatic tool for clinical evaluation of consciousness impairment [30], serving as a cornerstone in defining SAE [6]. Even subtle alterations in mental status, such as GCS scores ranging from 13 to 14, independently correlate with mortality [31]. The prognosis of SAE may be intertwined with impaired brain autoregulation and inadequate cerebral perfusion. Continuous monitoring of cerebral blood oxygen levels via near-infrared spectroscopy enables delineation of the blood pressure range conducive to optimizing brain autoregulation, thereby enhancing cerebral perfusion in SAE [32]. The threshold for detrimental cerebral hypoperfusion manifests variably across individuals, and dysregulation in SAE may arise from blood pressure dipping below the optimal range [32]. Our findings align with this notion, indicating a detrimental association between lower blood pressure and poor prognosis in SAE. Additionally, respiratory rate, a fundamental vital sign, emerges as a pivotal indicator in emergency and critical care settings, exhibiting robust prognostic value in sepsis patients [33].

In terms of laboratory parameters incorporated into our model, blood urea nitrogen serves as a reflective indicator of renal function, in common with urine output. Sonneville et al [31], in a multicenter retrospective study, identified acute renal failure as a risk factor for SAE, attributing this association to biological alterations potentially impacting brain function, including severe acidosis and uremia. Additionally, diminished renal function may precipitate the accumulation of neurotoxic substances [31]. Notably, elevated blood urea nitrogen levels [13] and diminished urine output emerge as risk factors for short-term mortality in SAE, a conclusion echoed in our study among the older adult population. Hemoglobin levels, commonly used to assess the overall anemia status across diverse patient populations, and an investigation involving 8853 patients with SAE revealed that the ratio of hemoglobin to red blood cell distribution width offers enhanced prognostic value for ICU all-cause mortality in patients with SAE [34]. Moreover, activated PTT waveform analysis has been posited as an effective means to discern sepsis [35], with previous data indicating that coagulation abnormalities exacerbate mortality risk in sepsis patients [36]. Elevated activated PTT levels, indicative of coagulation dysregulation, have been closely associated with SAE mortality [14], corroborated by our findings within the older adult population. Hypoxemia presents a prevalent concern among ICU patients, with its severity proving to be an independent predictor of mortality within this cohort [37]. Reduced oxygen saturation has emerged as a mortality risk factor in SAE [12], paralleling our assertion that diminished oxygenation index poses a risk to 28-day survival in older adult patients with SAE.

The type of initial care unit, AG, and body weight constitute 3 pivotal variables within our model. Previous studies have suggested that the introduction of specialized neurocritical care teams, including a dedicated full-time neurointensivist, is associated with significantly reduced in-hospital mortality and length of stay, although no effect was observed on readmission rates or long-term mortality [38]. Moreover, the presence of a neurointensivist has been linked to improved clinical outcomes, particularly among patients with aneurysmal subarachnoid hemorrhage, whereas patients with intracerebral hemorrhage often exhibit poor prognosis regardless of the availability of a neurointensive care unit or physician [39]. In our analysis, however, the type of ICU (including neurosurgical intensive care unit) did not appear to exert a strong influence on short-term mortality in older adult patients with SAE. However, existing mortality prediction models for SAE have not thoroughly explored the interplay between these factors, including the initial care unit type, and SAE [11-14], necessitating further inquiry in forthcoming high-quality investigations. Body weight influences lifespan, and notably, BMI exhibits a U-shaped relationship with 28-day mortality rates in severe sepsis patients [40]. Nevertheless, in prognosticating sepsis outcomes, the focus should extend beyond mere BMI-mortality associations, emphasizing the nuanced interplay with disease severity [41]. A prospective cohort study involving 862 older adult individuals aged 65 years and older in South Korea unveiled high serum AG as a potential independent predictor of overall mortality in this demographic, with elevated AG also impacting cardiovascular and infection-related mortality [42]. Lower body weight and elevated AG emerge as mortality risk factors in older adult SAE, as delineated in our investigation.

### Limitations

This study is subject to several inherent limitations warranting discussion. Primarily, despite the extensive clinical dataset available within the MIMIC-IV database, the presence of missing values and outliers poses a potential challenge. To address this concern, rigorous data cleaning and processing protocols were implemented to enhance data quality and mitigate potential biases. Second, certain pertinent clinical features, such as electroencephalogram data, central imaging techniques, cerebral oxygen levels, neurofilament light chain, and cerebral blood flow measurements, were not incorporated into our analysis due to their absence within the MIMIC database. Nevertheless, our study used rigorous inclusion and exclusion criteria to ensure well-defined cohorts. Moreover, this study focused on investigating a broad array of clinical features and hematological parameters that were readily accessible. Additionally, although variables such as lactate and white blood cell count were available, they were excluded during the LASSO regression variable selection process. Other potentially relevant inflammatory markers, including procalcitonin, C-reactive protein, serum amyloid A, and heparin-binding protein, were not included due to their unavailability in the database. To enhance the clinical practicality of the model, our study focused on commonly measured and readily accessible variables as candidate predictors. Third, our study solely undertook internal validation of the predictive model. Further external validation across diverse patient cohorts is imperative to ascertain the generalizability and robustness of the established nomogram. Lastly, the retrospective nature of this investigation underscores the imperative for future prospective studies to validate the clinical applicability and effectiveness of our developed nomogram. Prospective studies would afford opportunities to confirm the predictive accuracy of the nomogram in real-time clinical settings, thus enhancing confidence in its utility and informing clinical decision-making.

### Conclusions

In this study, we developed and internally validated a nomogram for predicting 28-day mortality in older adult patients with SAE using commonly available clinical data from the MIMIC-IV database. The model demonstrated satisfactory discriminative performance and calibration, with potential clinical utility as compared to traditional ICU scoring systems. Additionally, by implementing the nomogram as a web-based tool, we aimed to improve its accessibility and practical use in clinical settings. While promising, further external validation and prospective studies are necessary to confirm its broader applicability and effectiveness in diverse health care environments.

## Appendix

Multimedia Appendix 1 Visualization of the extent of missingness for each variable using the DataExplorer package in R.

Multimedia Appendix 2 Baseline characteristics of geriatric patients with sepsis-associated encephalopathy: a comprehensive overview of demographic and clinical features in the study cohort.

Multimedia Appendix 3 VIF values of included predictors for assessing collinearity. VIF: variance inflation factor.

Multimedia Appendix 4 ROC curves of the constructed nomogram versus conventional ICU scoring systems in the development and validation cohorts. ICU: intensive care unit; ROC: receiver operating characteristic.

## Abbreviations

AG: anion gap
APS III: Acute Physiology Score III
C-index: concordance index
GCS: Glasgow Coma Scale
ICD: International Classification of Diseases
ICU: intensive care unit
IDI: integrated discrimination improvement
LASSO: least absolute shrinkage and selection operator regression
MIMIC: Medical Information Mart for Intensive Care
MIMIC-IV: Medical Information Mart for Intensive Care IV
NRI: net reclassification improvement
OR: odds ratio
PaCO2: partial pressure of CO2
PTT: partial thromboplastin time
SAE: sepsis-associated encephalopathy
SOFA: sequential organ failure assessment
TRIPOD: Transparent Reporting of a Multivariable Prediction Model for Individual Prognosis or Diagnosis

## Data Availability

The original data are available online [16].
