# Supplementary figures and images for: Online Clinical Calculator for Predicting 28-Day Mortality in Older Adult Patients With Sepsis-Associated Encephalopathy: Retrospective Study Using MIMIC-IV
Source: JMIR Med Inform. 2025 Dec 4;13:e76417. doi: 10.2196/76417 (PMC12715468; doi:10.2196/76417)

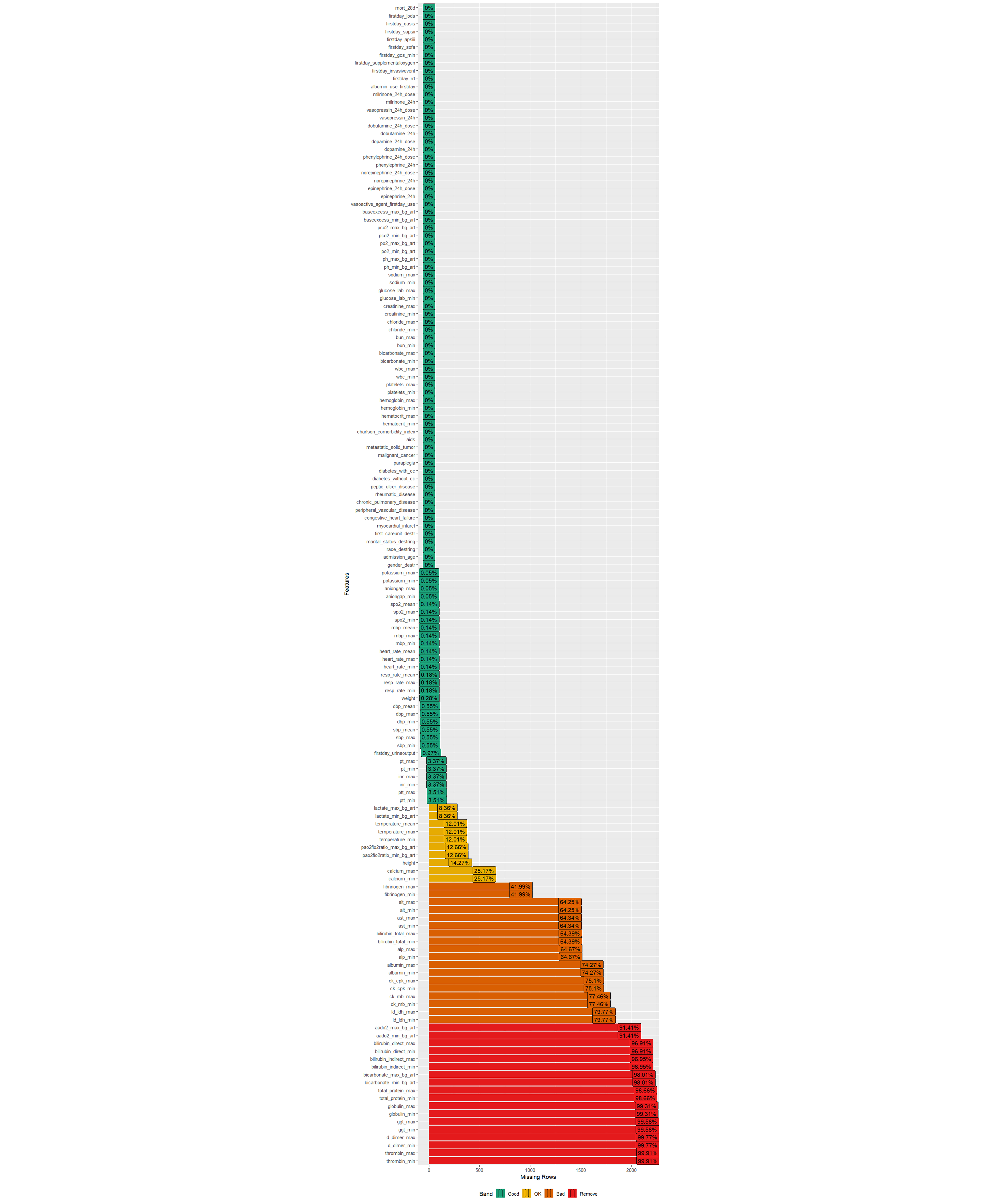

Supplement: Multimedia Appendix 1 [file medinform_v13i1e76417_app1.png]

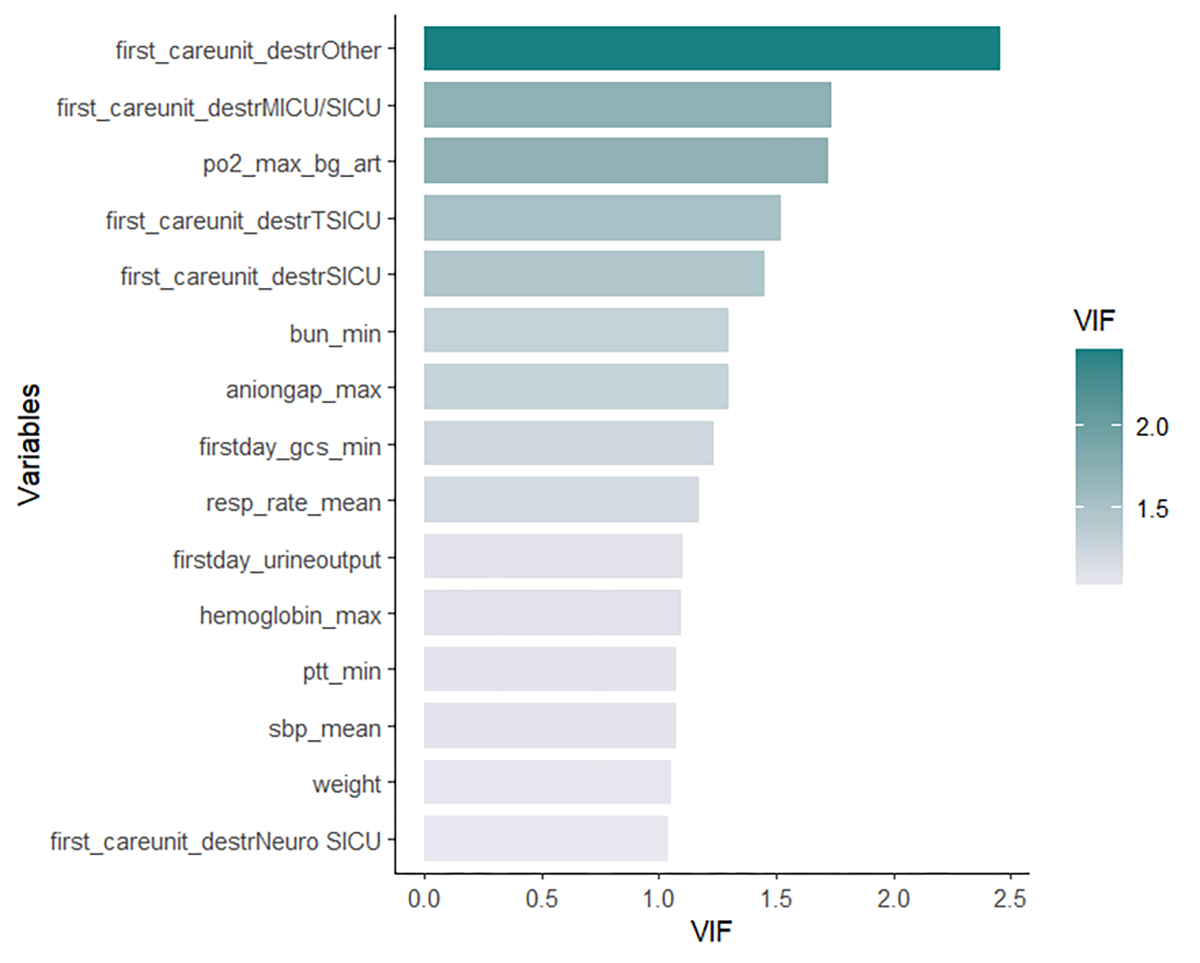

Supplement: Multimedia Appendix 3 [file medinform_v13i1e76417_app3.png]

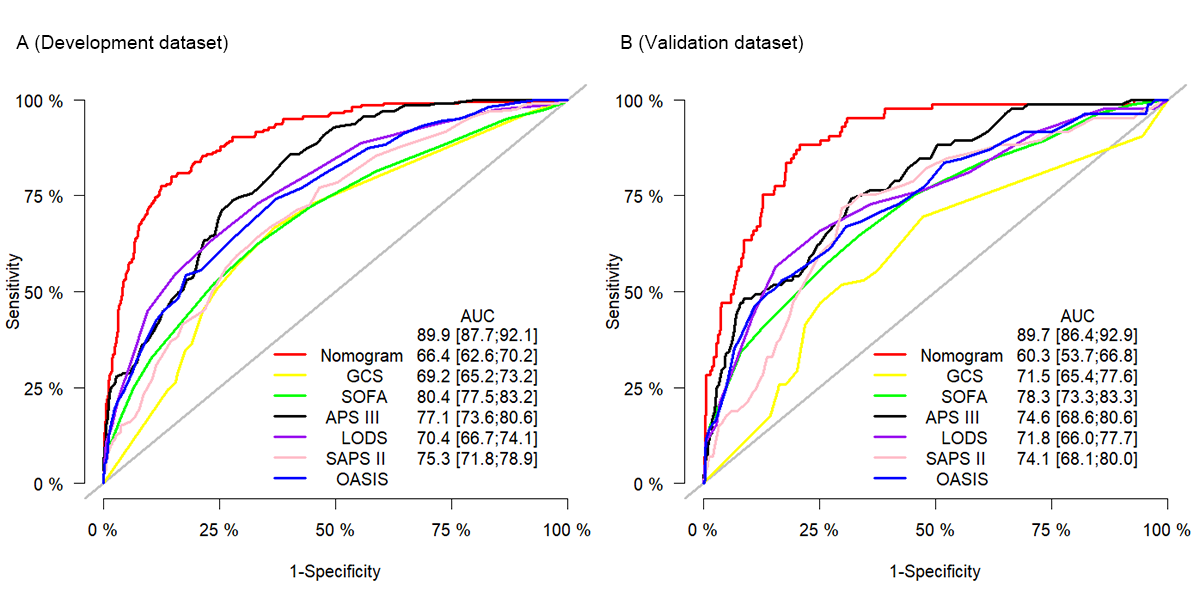

Supplement: Multimedia Appendix 4 [file medinform_v13i1e76417_app4.png]
