# Supplementary material for: Online Clinical Calculator for Predicting 28-Day Mortality in Older Adult Patients With Sepsis-Associated Encephalopathy: Retrospective Study Using MIMIC-IV
Source: JMIR Med Inform. 2025 Dec 4;13:e76417. doi: 10.2196/76417 (PMC12715468; doi:10.2196/76417)
Supplement: Multimedia Appendix 2 [file medinform_v13i1e76417_app2.doc]

| Characteristics | | All patients (N=2165) | | | Development dataset (n=1531) | | | Validation dataset (n=634) | | *P* value | |
| --- | --- | --- | --- | --- | --- | --- | --- | --- | --- | --- | --- |
| Age (years), median (IQR) | | 75.45 (70.19, 81.22) | | | 75.35 (70.30, 81.20) | | | 75.80 (70.10, 81.26) | | .82 | |
| Male, n (%) | | 1,247 (58%) | | | 886 (58%) | | | 361 (57%) | | .69 | |
| Weight (kg), median (IQR) | | 78.21 (66.80, 91.00) | | | 78.00 (66.45, 90.70) | | | 79.00 (67.10, 92.88) | | .37 | |
| **Race, n (%)** | | | | | | | | | | | |
|  | White | 1,598 (74%) | | | 1,133 (74%) | | | 465 (73%) | | .90 | |
|  | Hispanic | 33 (1.5%) | | | 21 (1.4%) | | | 12 (1.9%) | |
|  | Black | 78 (3.6%) | | | 55 (3.6%) | | | 23 (3.6%) | |
|  | Asian | 43 (2.0%) | | | 29 (1.9%) | | | 14 (2.2%) | |
|  | Other | 413 (19%) | | | 293 (19%) | | | 120 (19%) | |
| **Marital status, n (%)** | | | | | | | | | | | |
|  | Married | 1,167 (54%) | | | 840 (55%) | | | 327 (52%) | | .44 | |
|  | Single | 303 (14%) | | | 210 (14%) | | | 93 (15%) | |
|  | Widowed | 384 (18%) | | | 258 (17%) | | | 126 (20%) | |
|  | Divorced | 146 (6.7%) | | | 106 (6.9%) | | | 40 (6.3%) | |
|  | Other | 165 (7.6%) | | | 117 (7.6%) | | | 48 (7.6%) | |
| **First care unit,** **n (%)** | | | | | | | | | | | |
|  | MICU ^a^ | 208 (9.6%) | | | 137 (8.9%) | | | 71 (11%) | | .11 | |
|  | SICU ^b^ | 178 (8.2%) | | | 126 (8.2%) | | | 52 (8.2%) | |
|  | MICU/SICU | 218 (10%) | | | 146 (9.5%) | | | 72 (11%) | |
|  | Neuro SICU ^c^ | 11 (0.5%) | | | 5 (0.3%) | | | 6 (0.9%) | |
|  | TSICU ^d^ | 183 (8.5%) | | | 136 (8.9%) | | | 47 (7.4%) | |
|  | Other ICU | 1,367 (63%) | | | 981 (64%) | | | 386 (61%) | |
| **Underlying diseases,** **n (%)** | | | | | | | | | | | |
|  | Myocardial infarct | 497 (23%) | | | 358 (23%) | | | 139 (22%) | | .46 | |
|  | Congestive heart failure | 714 (33%) | | | 501 (33%) | | | 213 (34%) | | .69 | |
|  | Chronic pulmonary disease | 667 (31%) | | | 470 (31%) | | | 197 (31%) | | .86 | |
|  | Diabetes without chronic complication | 479 (22%) | | | 334 (22%) | | | 145 (23%) | | .59 | |
|  | Diabetes with chronic complication | 131 (6.1%) | | | 92 (6.0%) | | | 39 (6.2%) | | .90 | |
|  | Rheumatic disease | 105 (4.8%) | | | 68 (4.4%) | | | 37 (5.8%) | | .17 | |
|  | Metastatic solid tumor | 116 (5.4%) | | | 81 (5.3%) | | | 35 (5.5%) | | .83 | |
| **CCI ^e^, median (IQR)** | | 6 (5, 8) | | | 6 (5, 8) | | | 6. (5, 8) | | .91 | |
| **Vital indicators, median (IQR)** | | | | | | | | | | | |
|  | Heart rate (beats/min) ^f^ | 68.00 (60.00, 76.00) | | | 68.00 (60.00, 76.00) | | | 66.91 (60.00, 76.00) | | .19 | |
|  | Heart rate (beats/min) ^g^ | 98.00 (88.00, 111.00) | | | 98.00 (88.00, 111.00) | | | 97.00 (88.00, 111.00) | | .83 | |
|  | Heart rate (beats/min) ^h^ | 82.09 (75.44, 90.48) | | | 82.09 (75.82, 90.51) | | | 82.08 (74.35, 90.43) | | .32 | |
|  | SBP ^i^ (mmHg) ^f^ | 86.00 (78.00, 93.00) | | | 86.00 (79.00, 93.00) | | | 86.00 (78.00, 93.00) | | .77 | |
|  | SBP (mmHg) ^g^ | 143.00 (133.00, 156.00) | | | 143.00 (133.00, 155.00) | | | 144.00 (134.00, 157.00) | | .33 | |
|  | SBP (mmHg) ^h^ | 112.38 (105.90, 119.70) | | | 112.50 (105.75, 119.38) | | | 112.17 (106.47, 119.80) | | .47 | |
|  | DBP ^j^ (mmHg) ^f^ | 43.00 (38.00, 47.00) | | | 43.00 (38.00, 47.00) | | | 42.00 (37.00, 47.00) | | .20 | |
|  | DBP (mmHg) ^g^ | 75.00 (67.00, 87.00) | | | 75.00 (67.00, 87.00) | | | 76.00 (68.00, 87.00) | | .37 | |
|  | DBP (mmHg) ^h^ | 56.09 (51.24, 61.21) | | | 56.09 (51.22, 61.45) | | | 56.17 (51.39, 60.64) | | .90 | |
|  | Temperature (°C) ^f^ | 36.22 (35.56, 36.56) | | | 36.20 (35.56, 36.55) | | | 36.28 (35.60, 36.56) | | .13 | |
|  | Temperature (°C) ^g^ | 37.30 (36.94, 37.80) | | | 37.30 (36.94, 37.78) | | | 37.33 (37.00, 37.80) | | .08 | |
|  | Temperature (°C) ^h^ | 36.75 (36.48, 37.05) | | | 36.75 (36.47, 37.04) | | | 36.75 (36.50, 37.10) | | .13 | |
|  | Heart rate (beats/min) ^f^ | 68.00 (60.00, 76.00) | | | 68.00 (60.00, 76.00) | | | 66.91 (60.00, 76.00) | | .19 | |
|  | Heart rate (beats/min) ^g^ | 98.00 (88.00, 111.00) | | | 98.00 (88.00, 111.00) | | | 97.00 (88.00, 111.00) | | .83 | |
|  | Heart rate (beats/min) ^h^ | 82.09 (75.44, 90.48) | | | 82.09 (75.82, 90.51) | | | 82.08 (74.35, 90.43) | | .32 | |
|  | Respiratory rate (breaths/min) ^f^ | 12.00 (9.00, 14.00) | | | 12.00 (9.00, 14.00) | | | 11.00 (9.00, 14.00) | | .58 | |
|  | Respiratory rate (breaths/min) ^g^ | 26.00 (23.00, 31.00) | | | 26.00 (23.00, 31.00) | | | 27.00 (23.00, 30.00) | | .72 | |
|  | Respiratory rate (breaths/min) ^h^ | 17.92 (16.12, 20.18) | | | 17.94 (16.12, 20.21) | | | 17.82 (16.13, 20.08) | | .92 | |
|  | Firstday GCS ^f k^ | 14.00 (9.00, 14.00) | | | 14.00 (9.00, 14.00) | | | 13.00 (9.00, 14.00) | | .80 | |
|  | Firstday urine output (L) | 1.58 (1.05, 2.29) | | | 1.59 (1.07, 2.32) | | | 1.55 (1.00, 2.26) | | .19 | |
| **Laboratory indicators, median (IQR)** | | | | | | | | | | | |
|  | Hematocrit (%) ^f^ | | | 27.10 (24.10, 30.90) | | | 27.20 (24.00, 30.90) | 27.10 (24.40, 31.28) | | .22 | |
|  | Hematocrit (%) ^g^ | | | 33.30 (30.60, 37.00) | | | 33.30 (30.60, 37.00) | 33.35 (30.50, 37.08) | | .92 | |
|  | Hemoglobin (g/L) ^f^ | | | 91.00 (80.00, 104.00) | | | 91.00 (80.00, 104.00) | 91.50 (81.00, 105.00) | | .26 | |
|  | Hemoglobin (g/L) ^g^ | | 110.00 (100.00, 122.00) | | | 110.00 (100.00, 122.00) | | | 111.00 (101.00, 122.00) | | .86 |
|  | Platelets (K/uL) ^f^ | 141.00 (106.00, 194.00) | | | 141.00 (106.00, 192.00) | | | 142.00 (107.00, 200.75) | | .60 | |
|  | Platelets (K/uL) ^g^ | 185.00 (143.00, 241.00) | | | 185.00 (144.00, 240.00) | | | 186.00 (143.00, 244.75) | | .98 | |
|  | White blood cells (K/uL) ^f^ | 9.90 (7.20, 12.90) | | | 9.80 (7.20, 12.75) | | | 10.00 (7.50, 13.00) | | .11 | |
|  | White blood cells (K/uL) ^g^ | 14.60 (11.10, 18.80) | | | 14.40 (10.90, 18.90) | | | 14.90 (11.50, 18.80) | | .22 | |
|  | Anion gap (mmol/L) ^f^ | 11.00 (10.00, 13.00) | | | 11.00 (10.00, 13.00) | | | 12.00 (10.00, 13.00) | | .91 | |
|  | Anion gap (mmol/L) ^g^ | 14.00 (12.00, 16.00) | | | 14.00 (12.00, 16.00) | | | 14.00 (12.00, 16.00) | | .74 | |
|  | Blood urea nitrogen (mg/dL) ^f^ | 6.07 (4.64, 9.28) | | | 6.07 (4.64, 9.28) | | | 6.07 (4.64, 9.19) | | .85 | |
|  | Blood Urea Nitrogen (mg/dL) ^g^ | 7.14 (5.36, 11.07) | | | 7.14 (5.36, 11.07) | | | 7.14 (5.36, 11.07) | | .99 | |
|  | Creatinine (μmmol/L) ^f^ | 79.56 (61.88, 106.08) | | | 79.56 (61.88, 106.08) | | | 79.56 (61.88, 106.08) | | .88 | |
|  | Creatinine (μmmol/L) ^g^ | 88.40 (70.72, 123.76) | | | 88.40 (70.72, 123.76) | | | 88.40 (70.72, 132.60) | | .88 | |
|  | Glucose (mmol/L) ^f^ | 6.06 (5.28, 7.00) | | | 6.11 (5.28, 7.00) | | | 6.00 (5.22, 6.89) | | .12 | |
|  | Glucose (mmol/L) ^g^ | 7.06 (6.06, 8.17) | | | 7.06 (6.06, 8.11) | | | 7.08 (6.00, 8.22) | | .60 | |
|  | Sodium (mEq/L) ^f^ | 137.00 (135.00, 139.00) | | | 137.00 (135.00, 139.00) | | | 137.00 (135.00, 139.00) | | .38 | |
|  | Sodium (mEq/L) ^g^ | 140.00 (138.00, 142.00) | | | 140.00 (138.00, 142.00) | | | 140.00 (138.00, 142.00) | | .15 | |
|  | PTT ^l^ (sec) ^f^ | 29.10 (26.50, 33.60) | | | 29.10 (26.40, 33.61) | | | 29.30 (26.70, 33.38) | | .69 | |
|  | PTT (mmol/L) ^g^ | 34.80 (29.80, 45.30) | | | 34.80 (29.80, 45.25) | | | 34.80 (29.90, 45.95) | | .77 | |
|  | PH ^f^ | 7.33 (7.28, 7.37) | | | 7.33 (7.28, 7.37) | | | 7.33 (7.28, 7.38) | | .48 | |
|  | PH ^g^ | 7.44 (7.40, 7.48) | | | 7.44 (7.40, 7.48) | | | 7.44 (7.40, 7.48) | | .09 | |
|  | PaO2 ^m^ (mmhg) ^f^ | 93.00 (75.00, 119.00) | | | 93.00 (75.00, 119.00) | | | 94.00 (75.25, 117.75) | | .48 | |
|  | PaO2 (mmhg) ^g^ | 356.00 (161.00, 429.00) | | | 358.00 (168.00, 430.00) | | | 347.50 (145.00, 424.00) | | .07 | |
|  | PaCO2 ^n^ (mmhg) ^f^ | 34.00 (31.00, 38.00) | | | 34.00 (31.00, 38.00) | | | 34.00 (31.00, 38.00) | | .91 | |
|  | PaCO2 (mmhg) ^g^ | 46.00 (41.00, 51.00) | | | 46.00 (41.00, 51.00) | | | 46.00 (41.00, 51.00) | | .48 | |
|  | Oxygenation index ^f^ | 199.00 (137.14, 266.10) | | | 200.00 (138.00, 266.38) | | | 196.90 (137.04, 265.83) | | .58 | |
|  | Oxygenation index ^g^ | 314.00 (231.00, 400.00) | | | 316.00 (232.50, 404.00) | | | 312.00 (227.50, 394.86) | | .23 | |
|  | Lactate (mmol/L) ^f^ | 1.30 (1.00, 1.70) | | | 1.30 (0.90, 1.70) | | | 1.30 (1.00, 1.72) | | .15 | |
|  | Lactate (mmol/L) ^g^ | 2.30 (1.50, 3.20) | | | 2.30 (1.50, 3.20) | | | 2.30 (1.50, 3.20) | | .62 | |
| **Intervention measures,** **n (%)** | | | | | | | | | | | |
|  | Invasive mechanical ventilation | 1,294 (60%) | | | | | 907 (59%) | 387 (61%) | | .44 | |
|  | Renal replacement therapy | 56 (2.6%) | | | | | 42 (2.7%) | 14 (2.2%) | | .48 | |
|  | Vasoactive agent use ^o^ | 1,501 (69%) | | | | | 1,062 (69%) | 439 (69%) | | .96 | |
|  | Albumin use | 44 (2.0%) | | | | | 27 (1.8%) | 17 (2.7%) | | .17 | |
| **Disease severity score, median (IQR)** | | | | | | | | | | | |
|  | Firstday SOFA ^p^ | 6.00 (5.00, 9.00) | | | 6.00 (5.00, 9.00) | | | 6.00 (5.00, 9.00) | | .53 | |
|  | Firstday APS III ^q^ | 47.00 (33.00, 70.00) | | | 46.00 (34.00, 70.50) | | | 48.00 (32.00, 69.75) | | .87 | |
|  | Firstday SAPS II ^r^ | 41.00 (34.00, 52.00) | | | 41.00 (34.00, 52.00) | | | 41.00 (34.00, 52.00) | | .41 | |
|  | Firstday OASIS ^s^ | 36.00 (30.00, 42.00) | | | 36.00 (30.00, 42.00) | | | 36.00 (30.00, 42.00) | | .47 | |
|  | Firstday LODS ^t^ | 6.00 (3.00, 8.00) | | | 5.00 (3.00, 8.00) | | | 6.00 (3.00, 8.00) | | .40 | |
| **Outcomes** | |  | | |  | | |  | |  | |
|  | 28-day mortality (%) | 290 (13.4%) | | | 205 (13.4%) | | | 85 (13.4%) | | .99 | |
|  | ICU mortality (%) | 212 (9.8%) | | | 146 (9.5%) | | | 66 (10%) | | .53 | |
|  | Hospital mortality (%) | 253 (12%) | | | 174 (11%) | | | 79 (12%) | | .47 | |
|  | ICU LOS (days) ^u^ | 3.13 (1.53, 6.00) | | | 3.09 (1.50, 5.59) | | | 3.22 (1.83, 6.41) | | .04 | |
|  | Hospital LOS (days) | 8.30 (5.74, 13.20) | | | 8.27 (5.77, 13.11) | | | 8.37 (5.73, 13.48) | | .73 | |

^a^ MICU: medical intensive care unit.

^b^ SICU: surgical intensive care unit.

^c^ Neuro SICU: neuro surgical intensive care unit.

^d^ TSICU: trauma surgical intensive care unit.

^e^ CCI: Charlson comorbidity index.

^f^ Represents the minimum values observed for key indicators on the initial day of admission to the ICU.

^g^ Signifies the maximum values attained for clinical parameters within the first 24 hours of ICU admission.

^h^ Denotes the mean values of relevant indicators recorded during the initial ICU admission day.

^i^ SBP: systolic blood pressure.

^j^ DBP: diastolic blood pressure.

^k^ GCS: Glasgow coma scale.

^l^ PTT: partial thromboplastin time.

^m^ PaO2: Partial pressure of oxygen.

^n^ PaCO2: Partial pressure of CO2.

^o^ Vasoactive agent was operationally defined by the administration of specific pharmacological agents—namely, norepinephrine, epinephrine, phenylephrine, dopamine, dobutamine, vasopressin, or milrinone—within the initial 24-hour period following admission to the ICU.

^p^ SOFA: Sequential Organ Failure Assessment.

^q^ APS III: Acute Physiology Score III.

^r^ SAPS II: Simplified Acute Physiology Score II.

^s^ OASIS: Oxford Acute Severity of Illness Score.

^t^ LODS: Logistic Organ Dysfunction System.

^u^ LOS: Length of Stay.
